# Effect of Sc and Zr microalloying on recrystallization behavior of 1xxx aluminum heat exchanger alloys during post-deformation annealing

**DOI:** 10.1186/s40712-025-00307-7

**Published:** 2025-06-23

**Authors:** Alyaa Bakr, Paul Rometsch, X.-Grant Chen

**Affiliations:** 1 Department of Applied Science, University of Quebec at Chicoutimi, Saguenay, QC G7H 2B1 Canada; 2 Arvida Research and Development Center, Rio Tinto Aluminium, Saguenay, QC G7S 4K8 Canada

**Keywords:** 1xxx aluminum alloys, Sc and Zr additions, Hot deformation, High-temperature annealing, Recrystallization

## Abstract

1xxx-series aluminum alloys are widely utilized in heat exchangers. During brazing, heat exchanger components are exposed to a short period of high temperature, which may trigger recrystallization and abnormal grain growth, ultimately compromising their mechanical properties. This study investigates the impact of Sc and Zr microalloying on the microstructure stability of hot deformed 1xxx alloys subjected to post-deformation annealing from 500 to 575 °C for 1 h to simulate brazing-type processes. Four alloys were studied: namely 1xxx base, Al-0.07Sc, Al-0.07Sc-0.10Zr and Al-0.19Sc-0.15Zr alloys. Annealing at 500 °C led to complete recrystallization in the base alloy, while higher annealing temperatures promoted abnormal grain growth. The Al-0.07Sc alloy resisted recrystallization at 500 °C but was fully recrystallized by 550 °C. In contrast, the Al-0.07Sc-0.10Zr alloy retained its grain stability up to 550 °C owing to the presence of stable Al_3_(Sc,Zr) precipitates; however, partial recrystallization occurred at 575 °C. The Al-0.19Sc-0.15Zr alloy preserved most of deformed microstructure even after annealing at 575 °C. It showed the highest recrystallization resistance among the four alloys studied owing to its highest number density and finest size of Al_3_(Sc,Zr) precipitates, which suggests that this alloy can be applied in even more extreme conditions including brazing temperatures above 575 °C.

## Introduction

Aluminum alloys are widely utilized in the automotive industry for heat exchangers, including those used in radiators, air conditioner evaporators, exhaust gas recirculation systems, and water and air charge cooling systems (Cole and Sherman 1995; Guía-Tello et al. 2020). The 1xxx alloys are considered ideal materials for heat exchanger tubes, owing to their high formability, excellent thermal conductivity, and superior corrosion resistance (Shakiba et al. 2015; Kim et al. 2020). Heat exchangers are typically fabricated by forming Al sheet and extruded tube stock into the required geometries, followed by brazing to assemble the components together (Benoit et al. 2020). Brazing sheets are often comprised of a core alloy and a clad layer. The former provides the strength and life cycle requirements, while the latter is usually a low melting point alloy (e.g., an Al-Si 4xxx alloy) that forms the brazed joints between the components of the heat exchanger (Miller et al. 2000). Maintaining microstructural stability during brazing is essential, as the elevated temperature can compromise the grain structure, often triggering abnormal grain growth (AGG) (Li et al. 2019a; Niu et al. 2019).

AGG is a microstructural phenomenon where certain grains grow extensively by consuming the surrounding finer recrystallized grains (Na et al. 2016; Humphreys and Hatherly 2012; Dennis et al. 2009; Liu et al. 2022). This results in an extremely inhomogeneous microstructure, which significantly compromises the properties (Payton et al. 2013). Abnormal grains exhibit higher grain boundary migration rates than their surrounding grains (Wang and Xu 2219). This enhanced boundary mobility can be attributed to several factors such as the crystallographic texture, variation in stored energy between grains, and uneven pinning by precipitates, where boundaries associated with certain grain orientations migrate more easily than others (Liu et al. 2022; Xu et al. 2196; Wang et al. 2011; Andersen et al. 1995). AGG has been shown to cause significant degradation in the strength of brazed heat exchanger tubes, as reported in several studies. For instance, Kraft (Kraft 2002) observed substantial grain growth during the brazing of AA1050 extruded heat exchanger tubes and reported a significantly lower strength in the internal wall of the tube compared to the bulk tube. Tang et al. (2018) and Li et al. (2019b) also reported a significant reduction in the pressure-bearing capacity of post-brazed AA1100 heat exchanger tubes owing to the AGG in the brazed microstructure. Given the detrimental impact of AGG on the mechanical integrity of brazed heat exchanger tubes, strategies to stabilize the grain structure during high-temperature exposure are crucial. One well-established strategy involves the use of fine and stable dispersoids within the Al matrix, which can effectively hinder subgrain boundary movement, suppress recrystallization, and restrict grain growth by exerting a pinning force on grain/subgrain boundaries (Guo et al. 2015; Wang et al. 2023; Li et al. 2013; Birol 2014; Lai et al. 2014; Algendy et al. 2023).

Among the various alloying strategies, microalloying with Sc has proven to be effective in enhancing the strength and the recrystallization resistance in aluminum alloys through the formation of thermally stable Al_3_Sc precipitates that inhibit grain boundary motion during high temperature exposures (Røyset and Ryum 2005a; Seidman et al. 2002; Davydov et al. 2000). The low lattice mismatch between L1_2_-Al_3_Sc precipitates and the matrix creates a coherent interface, further strengthening the alloy and stabilizing its grain structure (Xu et al. 2019; Luca et al. 2016). These precipitates exert a drag force on grain and sub-grain boundaries, inhibiting the nucleation and growth of recrystallized grains (Forbord et al. 2008; Yan et al. 2021). Despite efforts to reduce production costs, Sc remains an expensive alloying element owing to supply limitations and complex extraction processes (Sverdrup and Sverdrup 2024; Phoung et al. 2030). A more economical alloying strategy involves co-additions with Zr that promote the formation of Al_3_(Sc, Zr) precipitates. These precipitates, featuring an Al_3_Sc core with a Zr-enriched shell, offer an even more enhanced resistance to thermal coarsening (Buranova et al. 2017; Knipling et al. 2010, 2011).

Several studies have demonstrated that Sc/Zr-containing precipitates are highly effective in regulating recovery, recrystallization, and grain growth in Al alloys, thus stabilizing their microstructures during high temperature exposure. For instance, Jia et al. (2012) reported that a cold-rolled Al-0.1Sc-0.11Zr (wt.%) alloy showed no recrystallization when isothermally annealed at 250 °C, and that the recrystallization was initiated only at 550 °C. Similarly, Ocenasek et al. (2001) observed that Al_3_(Sc,Zr) precipitates stabilized the deformed microstructure at high annealing temperatures, and the results showed that an Al–Mg alloy with 0.25 wt.% Sc and 0.08 wt.% Zr resisted recrystallization after annealing at 520 °C for 8 h, with partial recrystallization near 600 °C. In another study by Babaniaris et al. (2020), it was reported that a cold-worked Al–Mg-Si alloy containing 0.25 wt.% Sc and 0.08 wt.% Zr exhibited strong resistance to recrystallization during annealing, remaining non-recrystallized after 8 h at 520 °C. Algendy et al. (2024) found that after the addition of 0.10 wt.% Sc + 0.08 wt.% Zr in Al–Mg-Mn 5083 rolled alloys, the rolled material retained a deformed fibrous structure during annealing at 400 °C for 30 min, with only 3.4% recrystallized grains. Lai et al. (2024) reported that the addition of 0.089 wt.% Sc and 0.10 wt.% Zr significantly improved the recrystallization resistance of an Al-5 Mg-3Zn rolled alloy, reducing the recrystallized fraction after solution treatment at 465 °C for 30 min from nearly 100% in the unmodified alloy to just 9% in the microalloyed variant. Li et al. (2014) showed that solution heat treatment at 470 °C for 1 h led to full recrystallization in an Al-Zn-Mg-Mn rolled alloy, while the addition of 0.24 wt.% Sc and 0.12 wt.% Zr suppressed recrystallization. Ye et al. (2023) also observed that the recrystallized fraction dropped sharply from 99.8% in the Al–Zn–Mg–Cu rolled alloy free Sc/Zr to just 12.6% in the Sc/Zr-modified alloy after solution treatment at 470 °C for 1 h. Lu et al. (1013) observed that following a welding thermal cycle, the 7055 alloy with only 0.12 wt.% Zr underwent extensive recovery and recrystallization. In contrast, the 7055 alloy containing 0.22 wt.% Sc and 0.11 wt.% Zr significantly enhanced microstructural stability, preserving 51% of its deformed structure. Xiao et al. (2020) observed that Al_3_(Sc,Zr) particles in an Al–Zn–Mg–Cu alloy with 0.07 wt.% Sc and 0.07 wt.% Zr remained stable after extrusion and solution heat treatment, promoting an elongated grain structure. Despite the strong recrystallization resistance demonstrated across various Al alloy systems, those studies mainly focused on non-1xxx series alloys. Moreover, they lacked specific emphasis on brazing or simulated brazing conditions. As a result, the behavior of Sc and Zr microalloying in 1xxx alloys under thermal exposures relevant to brazed heat exchangers remains largely underexplored.

This study aims to evaluate the impact of Sc and Zr microalloying on deformed microstructures of 1xxx alloys, particularly in scenarios involving short thermal exposures from 500 to 575 °C to simulate the brazing process. While conventional aluminum brazing is typically performed at ~ 600 °C, recent developments in filler metal design have aimed to lower the brazing temperature to mitigate abnormal grain growth. Several studies have proposed low-melting-point filler metals with adequate bonding strength having melting ranges as low as 500–526 °C. These include Al-7Si-20Cu-2Sn-1 Mg (Chuang et al. 2000), Zn-15Al (Zhao et al. 2018), and modified Al-12Si alloys containing Cu and Sn (Tsao et al. 2001, 2002). The selected thermal exposure temperatures between 500 and 575 °C in this study reflect potential brazing conditions enabled using recently developed low-melting-point filler metals. The hot deformation behavior is assessed through uniaxial hot compression testing, while the microstructural evolution during hot deformation and post-deformation annealing is analyzed using electron backscatter diffraction (EBSD) and transmission electron microscopy (TEM) to assess the effectiveness of the Sc/Zr-containing precipitates in enhancing the thermal stability and recrystallization resistance.

### Experimental procedure

Four experimental 1xxx alloys with and without Sc and Sc + Zr additions were investigated. The alloys were prepared from commercially pure aluminium (99.85%), Al-25%Fe, Al-50%Si, Al-5%Ti-1%B, Al-2%Sc, and Al-15%Zr master alloys (wt.%). Approximately 5 kg of each material was batched in an electrical resistance furnace and cast at 720 °C in a rectangular permanent steel mold with dimensions of 30 × 40 × 80 mm. The chemical compositions of the four alloys, analyzed by optical emission spectroscopy (Thermo ARL 4460), are listed in Table 1. All cast ingots were subjected to a low-temperature homogenization treatment at 350 °C for 24 h with a heating rate of 50 °C/h to promote Al_3_Sc/Al_3_(Sc,Zr) precipitation (Røyset and Ryum 2005b; Na et al. 2021; Su et al. 2025; Huang et al. 2015a, 2015b).

**Table 1:** Chemical compositions of the experimental alloys (wt.%)

Alloy	Si	Fe	Ti	Sc	Zr	Al
Base alloy	0.12	0.17	0.02	--	--	Bal.
Al-0.07Sc	0.13	0.23	0.02	0.07	--	Bal.
A-0.07Sc-0.1Zr	0.15	0.12	0.02	0.07	0.10	Bal.
Al-0.19Sc-0.15Zr	0.12	0.19	0.02	0.19	0.15	Bal.

Hot compression tests were conducted using a Gleeble 3800 thermomechanical testing unit. Cylindrical samples 10 mm in diameter and 15 mm in height were machined from the homogenized ingots. Hot compression tests were carried out at 450 °C, reflecting typical industrial extrusion conditions for aluminum heat exchanger alloys (Li et al. 2019a; Tang et al. 2018, 2014; Lee et al. 2016; Fang et al. 2020). A constant strain rate of 1 s^−1^ was applied. Each specimen was heated to 450 °C at a rate of 2 °C/s and held for 2 min, and then compressed to a true strain of 0.8. At least three repetitions were conducted to ensure reproducibility and reliability. After deformation, the sample was water-quenched to preserve the deformed microstructure. To evaluate the recrystallization behavior of the different alloys, post-annealing treatments were conducted at 500 °C, 550 °C, and 575 °C for 1 h. The selected thermal exposure temperatures correspond to niche brazing scenarios made possible using the new developed low-melting-point filler alloys (Chuang et al. 2000; Zhao et al. 2018; Tsao et al. 2001, 2002).

Microstructural characterization of the alloys was performed using an optical microscope (OM, Nikon-Eclipse ME600), a scanning electron microscope (SEM, JEOL 6480LV) and a transmission electron microscope (TEM, JEOL JEM-2100). EBSD analysis was conducted on samples sectioned parallel to the compression axis. EBSD maps, with a step size of 1 μm, were used to reveal sub-grain structures and misorientation distributions. TEM analysis, performed at 200 kV, was used to observe the Al_3_Sc/Al_3_(Sc,Zr) precipitates. TEM samples were prepared using a twinjet electropolishing unit, operated at 15 V and − 30 °C, in an electrolyte solution of 30% nitric acid and 70% methanol. Observations of precipitates were made in the dark-field mode near the [001] zone axis.

The size and number density of the spherical Al_3_Sc/Al_3_(Sc,Zr) precipitates were measured using ImageJ image analysis software on several TEM images. The number density *N*_*d*_ was calculated using the counted number of precipitates *N* in an area of *A* in the TEM images using Eq. 1 (Qian et al. 2018).

1 $$N_d=\frac N{A\ast(D+t)}$$

where *D* is the average diameter of the precipitates, and *t* is the thickness of the TEM sample measured using convergent-beam electron diffraction.

## Results

### As-homogenized microstructures

**Fig. 1:**
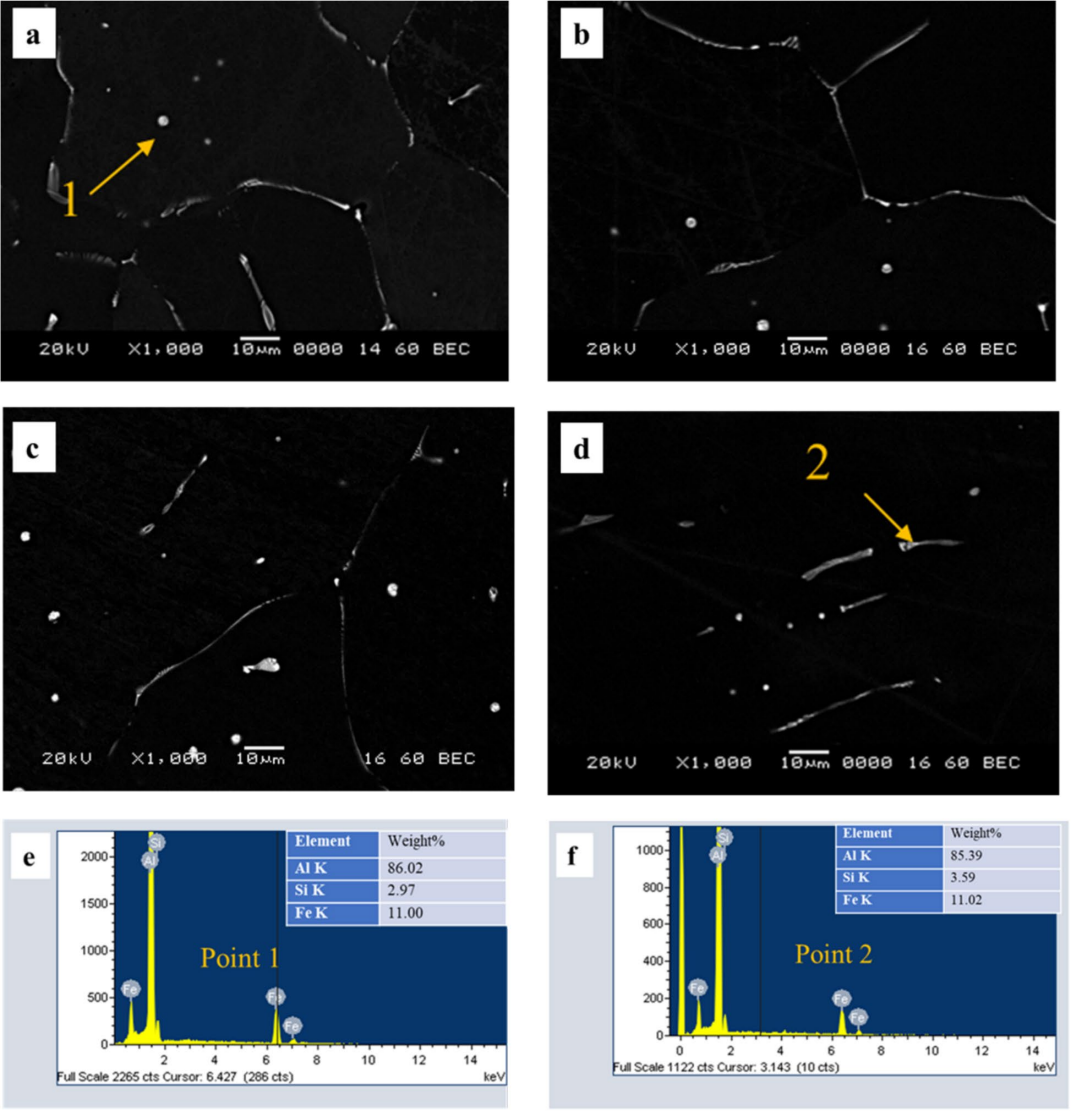
**a**–**d** SEM images of as-homogenized microstructures, **a** base, **b** Al-0.07Sc, **c** Al-0.07Sc-0.10Zr, and **d** Al-0.19Sc-0.15Zr alloys. **e**, **f** corresponding SEM–EDS point analysis of Fe-rich intermetallic phases observed in **a** and **d**

Figure 1 displays the SEM micrographs of as-homogenized microstructures of the four investigated alloys. All four alloys exhibited similar microstructures, composed of α-Al matrix and Fe-rich intermetallic particles appearing in both Chinese-script and compact forms distributed along Al dendrite boundaries. The main Fe-rich intermetallic phase was Al_8_Fe_2_Si identified by SEM–EDS analysis (see Fig. 1e, f). A similar Fe-rich intermetallic phase has been reported in aluminum 1xxx alloys with comparable levels of Fe and Si (Zhao et al. 2015; Shakiba et al. 2014). In the SEM images, no primary Al_3_(Sc) or Al_3_(Sc,Zr) intermetallic particles were found in the as-homogenized microstructures of the three Sc-containing alloys. This observation aligns with literature recommendations maintaining Sc at low levels of < 0.30 wt.% to promote the formation of fine Al_3_(Sc,Zr) dispersoids while minimizing the risk of primary intermetallic formation (Davydov et al. 2000). Knipling et al. (2010) demonstrated that dilute Al-Sc and Al-Zr alloys exhibited no primary Sc/Zr-containing intermetallic phases in the as-cast state when processed under comparable composition ranges. Similarly, Deng et al. (2019) reported the absence of primary Al₃(Sc₁₋ₓZrₓ) particles in an Al–Mg–Mn alloy containing 0.10 wt.% Sc and 0.12 wt.% Zr.

**Fig. 2:**
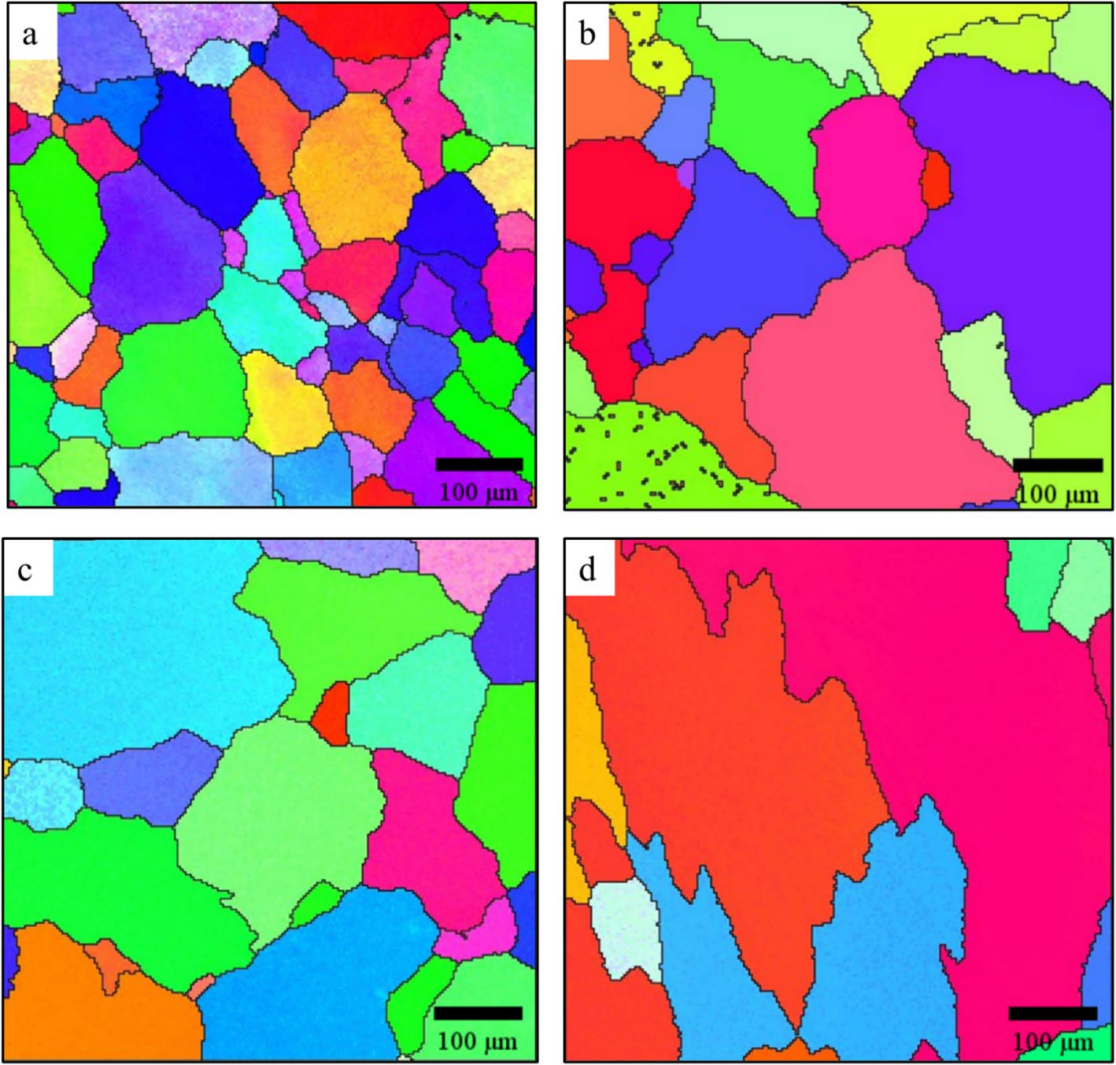
EBSD Maps of the as-homogenized grain structures **a** base, **b** Al-0.07Sc, **c** Al-0.07Sc-0.10Zr, and **d** Al-0.19Sc-0.15Zr alloy

Figure 2 presents the EBSD maps of the as-homogenized grain structures of the four investigated alloys. The base alloy showed an average equivalent grain diameter of 72.5 µm, which increased to 106.8 µm in the Al-0.07Sc alloy. This trend aligns with the previous findings by Huang et al. (2020), who reported that trace Sc addition diminished the grain refining effectiveness on Al-Ti-B grain refiners. Deng et al. (2012) observed that a low Sc addition of 0.1 wt.% in an Al–Zn–Mg alloy did not refine the grains, whereas the refinement was only achieved at 0.25 wt.% Sc. These results support the understanding that sub-eutectic Sc levels are insufficient to induce nucleation-based grain refinement (Hyde et al. 2001). The equivalent grain diameters in the Al-0.07Sc-0.1Zr and Al-0.19Sc-0.15Zr alloys were 111.2 and 134.8 µm, respectively. This grain coarsening can be attributed to the poisoning effect of Zr, as reported by Bunn et al. (1999). They observed that Zr substitutes for Ti in TiB₂ grain refiners, thereby reducing their effectiveness.

### Precipitation of Al_3_Sc and Al_3_(Sc,Zr) during homogenization

**Fig. 3:**
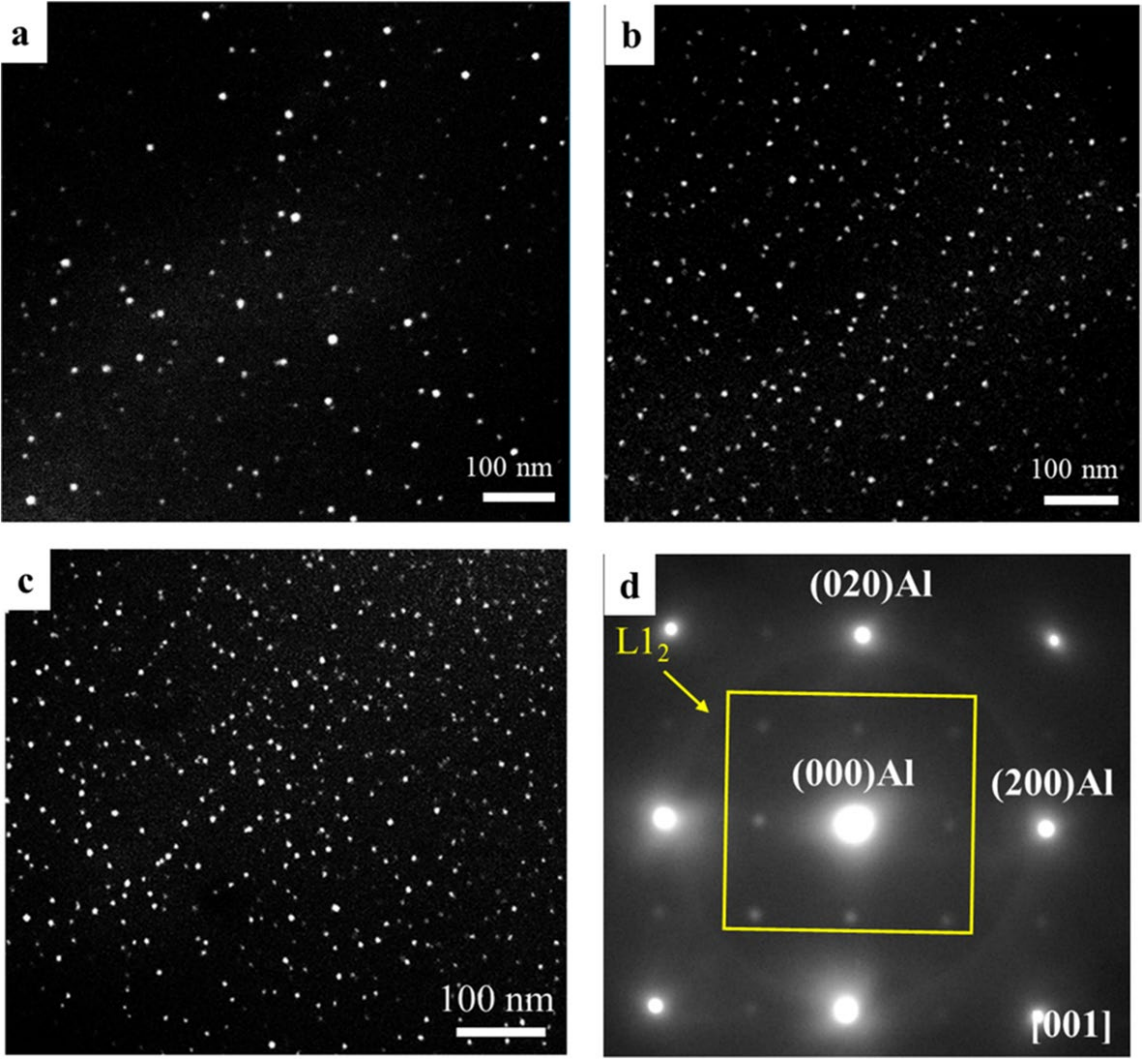
Dark-field TEM images of **a** Al-0.07Sc, **b** Al-0.07Sc-0.10Zr, and **c** Al-0.19Sc-0.15Zr alloys after homogenization at 350 °C for 24 h, and **d** SAED pattern of the Al-0.19Sc-0.15Zr alloy

**Fig. 4:**
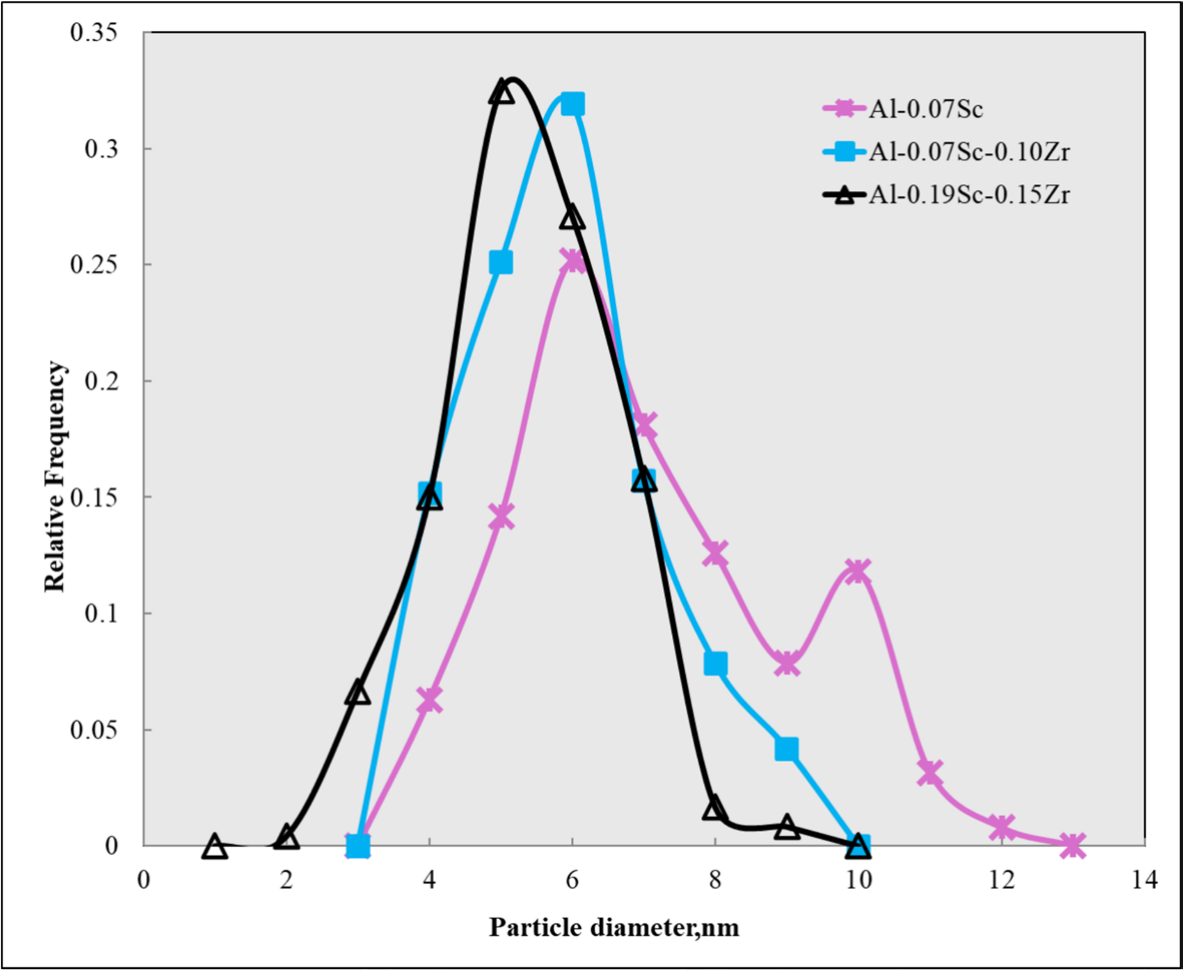
Histograms of the precipitate size distributions for the three Sc-containing alloys after homogenization at 350 °C

Figure 3a–c shows the dark-field TEM images of Al_3_Sc and Al_3_ (Sc, Zr) nanoprecipitates in the three Sc-containing alloys after low-temperature homogenization. Figure 3d shows a typical selected area electron diffraction (SAED) pattern obtained along the [001] zone axis, displaying faint spots from the precipitates between the α-Al spots and confirming that the precipitates have an L1₂ structure (Dorin et al. 2018). Figure 4 illustrates the size distribution of the Al_3_Sc and Al_3_(Sc,Zr) precipitates for the three Sc-containing alloys. Increasing the Sc and Zr content affected the average size and size range of the Al_3_Sc and Al_3_(Sc,Zr) precipitates. For instance, the Al-0.07 Sc alloy showed a broad size distribution, while the Al-0.07Sc-0.1Zr and Al-0.19Sc-0.15Zr alloys exhibited narrower size distributions. The quantitative results of the precipitate characteristics in the three Sc-containing alloys are displayed in Table 2. With increasing contents of Sc and Zr, the average size (D) of the precipitates decreased and the number density (Nd) increased. The lower diffusivity of Zr in aluminum, compared to that of Sc, leads to its delayed incorporation into precipitates, resulting in a Zr-rich shell that surrounds an Al_3_Sc core. This core–shell structure contributes to a slower coarsening rate of Al_3_(Sc,Zr) precipitates and results in smaller precipitate sizes and higher number densities (Tolley et al. 2005; Fuller and Seidman 2005).

**Table 2:** Quantitative measurements of Al_3_Sc/Al_3_(Sc,Zr) precipitates after homogenization

Alloy	Average *D* (nm)	Number density *N*_d_ (10 ^21^ m^−3^)
Al-0.07Sc	6.6±1.9	3.2±0.5
Al-0.07Sc-0.10Zr	5.5±1.3	7.2±0.2
Al-0.19Sc-0.15Zr	4.8±1.2	9.0±0.7

### Effect of Sc and Zr on hot deformation

**Fig. 5:**
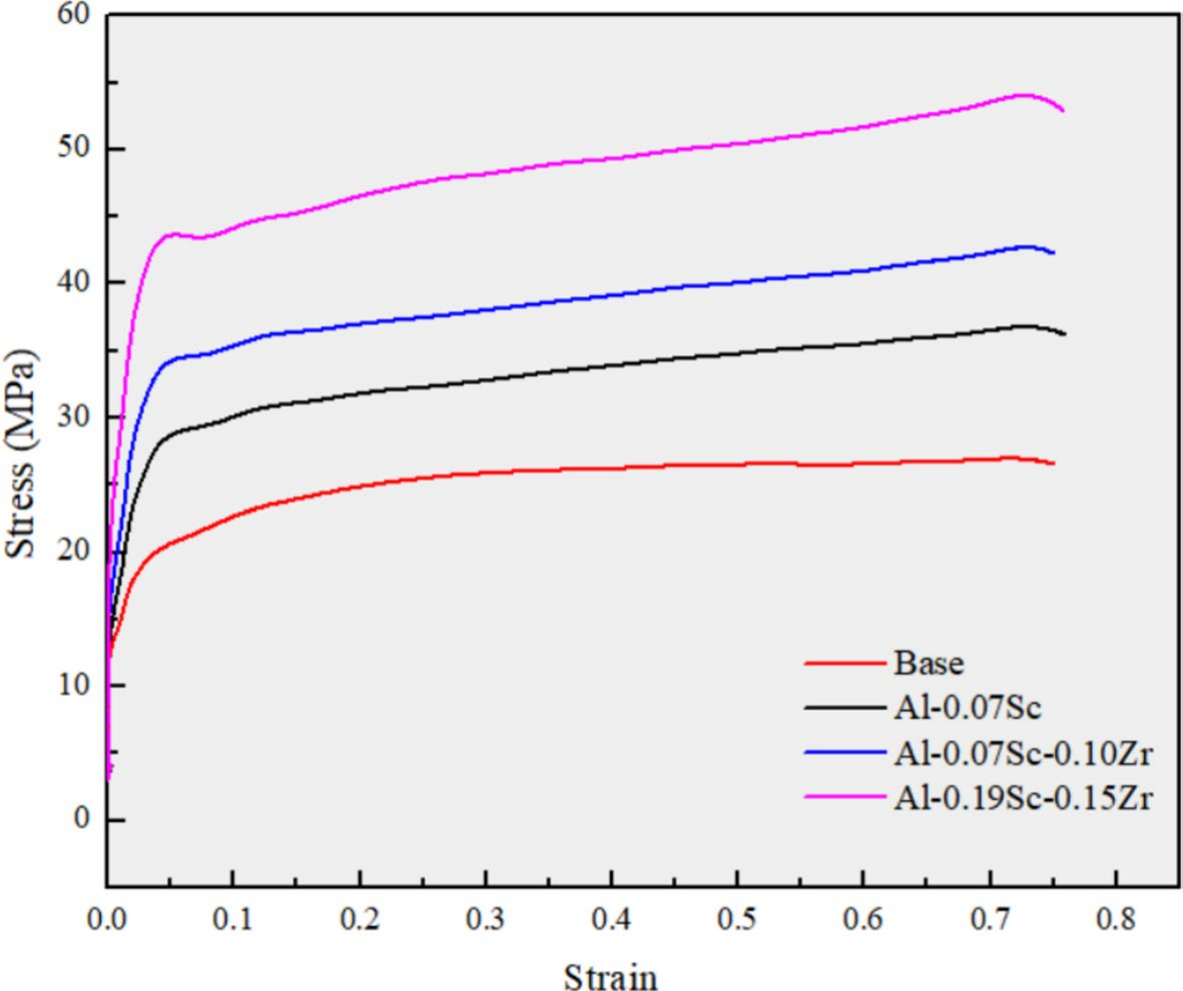
True stress–strain curves during hot compression deformation at 450 °C and 1 s^−1^ for the four experimental alloys

Figure 5 shows the true stress–strain curves during hot compression deformation of the four alloys studied. The flow behavior of the alloys exhibited typical characteristics of high-temperature deformation, where dynamic recovery (DRV) was the dominant softening mechanism (Mcqueen et al. 2016). Following the initial elastic response, the stress increased rapidly owing to dislocation multiplication and then continued to rise steadily as dynamic recovery progressed (McQueen and Ryan 2002). The flow stress of the base alloy exhibited a near plateau after the initial deformation stage (at ~ 0.2 strain), indicating a balance between the work hardening and dynamic recovery during further deformation. In contrast, all three Sc-containing alloys showed a continuous increase in flow stress throughout the entire hot compression process. The addition of 0.07 wt.% Sc increased the flow stress from 27 MPa for the base alloy to 36.5 MPa. The combined addition of 0.07 wt.% Sc and 0.10 wt.% Zr further raised the flow stress to 42.3 MPa. The Al-0.19Sc-0.15Zr alloy exhibited the highest flow stress of 53.5 MPa. The higher flow stresses in the Al-Sc and Al-Sc-Zr alloys are attributed to the presence of numerous Al_3_Sc and Al_3_(Sc,Zr) precipitates in the Al matrix (Fig. 3), which exert a retardation effect on DRV (Babaniaris et al. 2020).

#### Microstructures after hot deformation

**Fig. 6:**
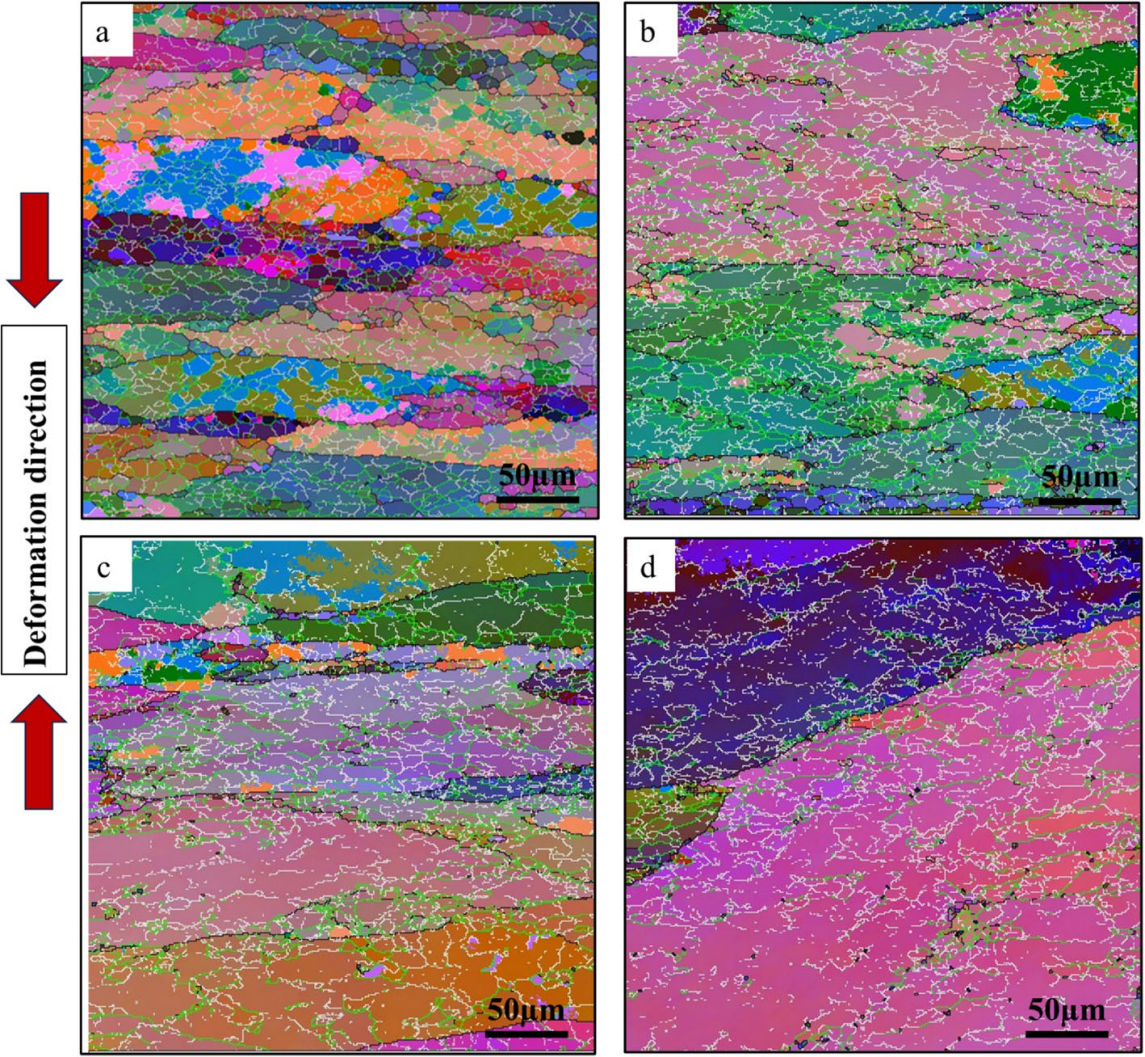
All Euler EBSD maps of **a** base alloy, **b** Al-0.07Sc, **c** Al-0.07Sc-0.10Zr, and **d** Al-0.19Sc-0.15Zr alloys after hot deformation, where the white, green, and black lines correspond to misorientation angles of 2–5°, 5–15°, and > 15°, respectively

**Fig. 7:**
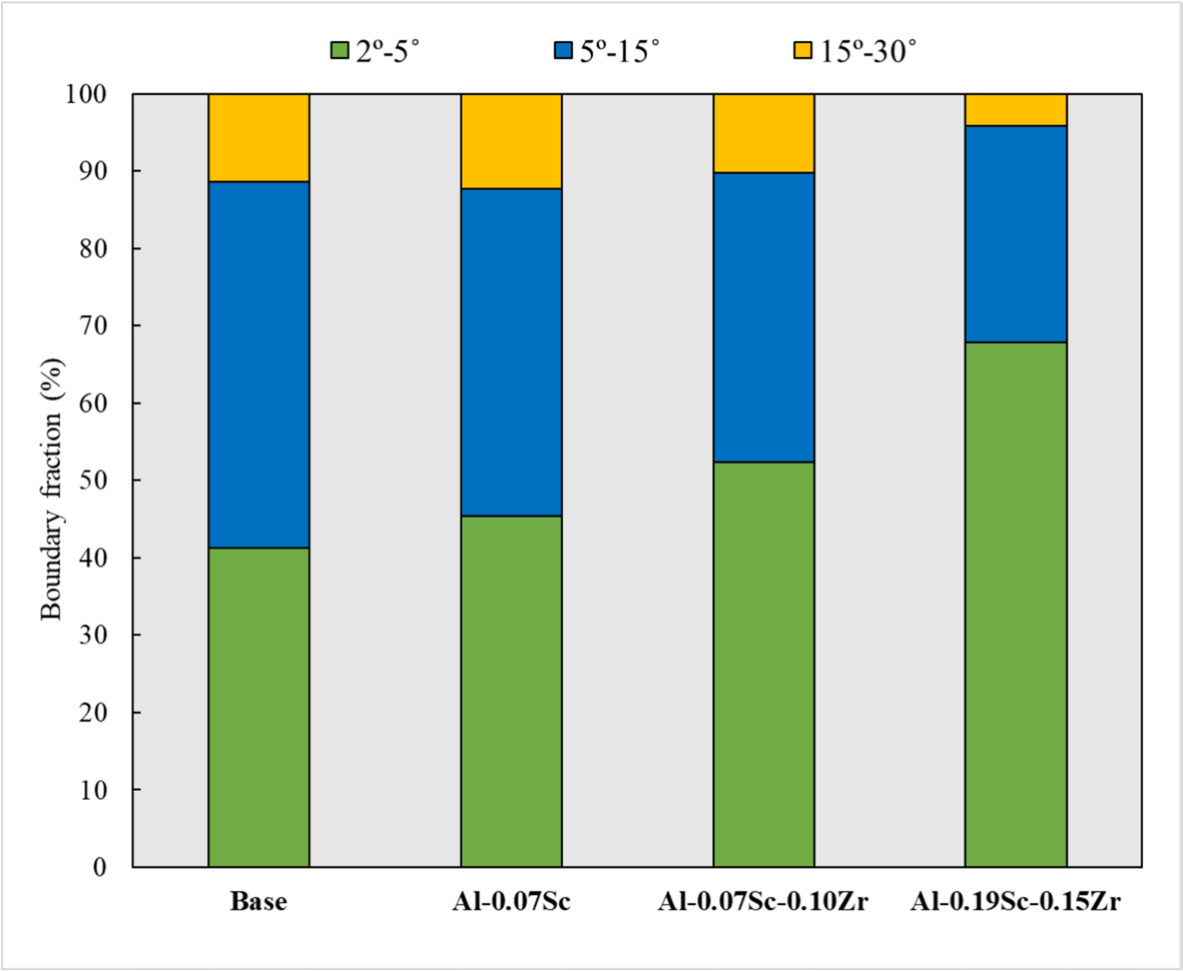
Evolution of misorientation angle distribution of boundaries after hot deformation for the four alloys studied

Figure 6 shows the All-Euler EBSD maps of the four investigated alloys, revealing the characteristics of the as-deformed grain structures. The grain structures of all four alloys were primarily composed of low-angle grain boundaries (LAGBs, 2º−5º) and medium-angle boundaries (MAGBs, 5º−15º), indicating that the alloys underwent dynamic recovery during deformation. The original grains were elongated perpendicular to the compression direction, and the as-deformed microstructure displayed a high density of sub grains. Figure 7 presents the misorientation angle distributions of grain/sub grain boundaries after hot deformation for the four alloys. Compared to the base alloy, the three Sc-containing alloys showed higher fractions of LAGBs. The percentages of boundaries with misorientations between 15º and 30º were similar across all four alloys, but significant differences were observed in the misorientation ranges of 2º–5º and 5º–15º. The fraction of LAGBs increased from 41.3% in the base alloy to 45.4% in the Al-0.07Sc alloy, and further to 52.3% and 67.9% in the Al-0.07Sc-0.10Zr and Al-0.19Sc-0.15Zr alloys, respectively. Conversely, the fraction of MAGBs decreased progressively, from 47.3% in the base alloy to 42.3% in the Al-0.07Sc alloy, and further to 37.4% in the Al-0.07Sc-0.1Zr alloy, with the lowest value of 27.9% observed in the Al-0.19Sc-0.15Zr alloy. The shift in the misorientation angle distributions for LAGBs and MAGBs indicates that the microalloying with Sc or Sc and Zr imposes greater restrictions on dynamic recovery (DRV) (Shi and Chen 2014).

**Fig. 8:**
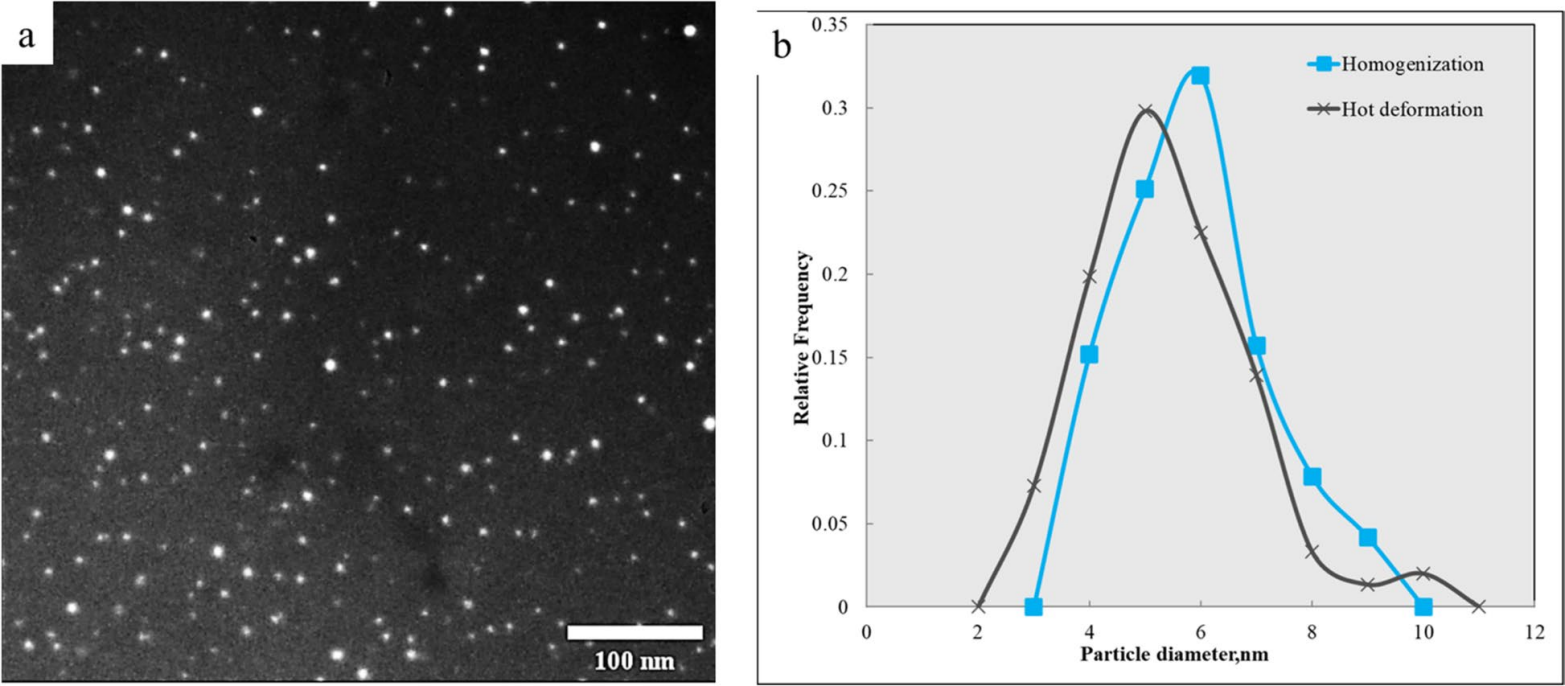
**a** Dark-field TEM image of Al_3_(Sc,Zr) precipitates in the Al-0.07Sc-0.10Zr alloy after the hot deformation. **b** Histogram of the precipitate size distributions

To further examine the evolution and stability of dispersoids during processing, TEM analysis was performed on the as-deformed Al-0.07Sc-0.10Zr alloy. Figure 8a shows a dark-field TEM image of the Al_3_(Sc,Zr) precipitates after hot deformation. The precipitates are uniformly distributed in the Al matrix and have an average size of 5.0 ± 1.4 nm ranging from 2.7 to 9.9 nm (Fig. 8b). Histograms of the precipitate size distributions after homogenization and after hot deformation are quite close. This suggests that Al_3_(Sc,Zr) precipitates, which formed during homogenization, remained almost unchanged and were thermally stable without coarsening during hot deformation. The fine, coherent nanoprecipitates play a critical role in impeding dislocation motion and delaying the recovery process, contributing to a higher flow stress, and improved microstructural stability during hot deformation (Babaniaris et al. 2020).

#### Effect of Sc and Zr on recrystallization behavior during post-deformation annealing

**Fig. 9:**
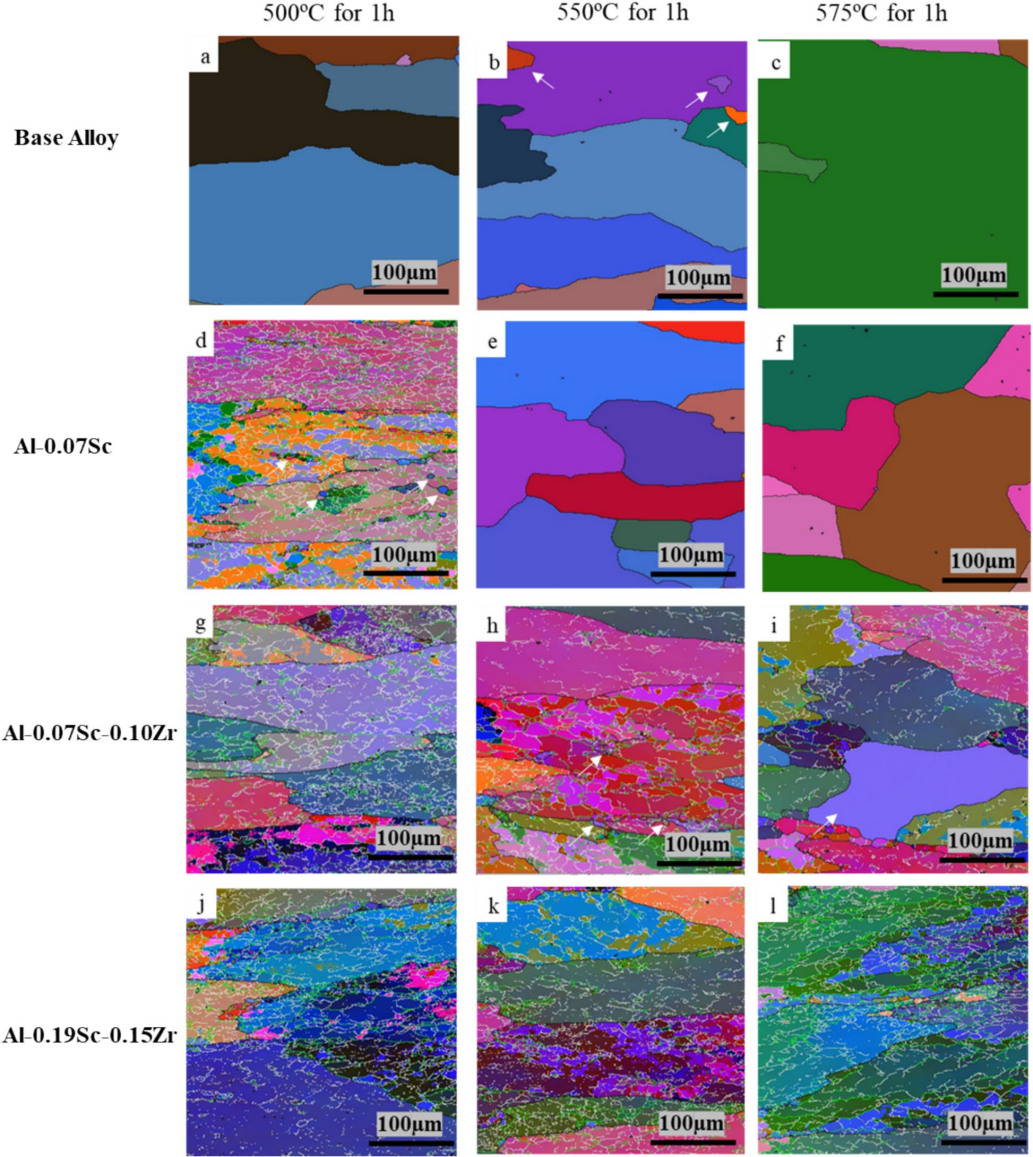
All Euler EBSD maps of the four studied alloys after post-deformation annealing

**Fig. 10:**
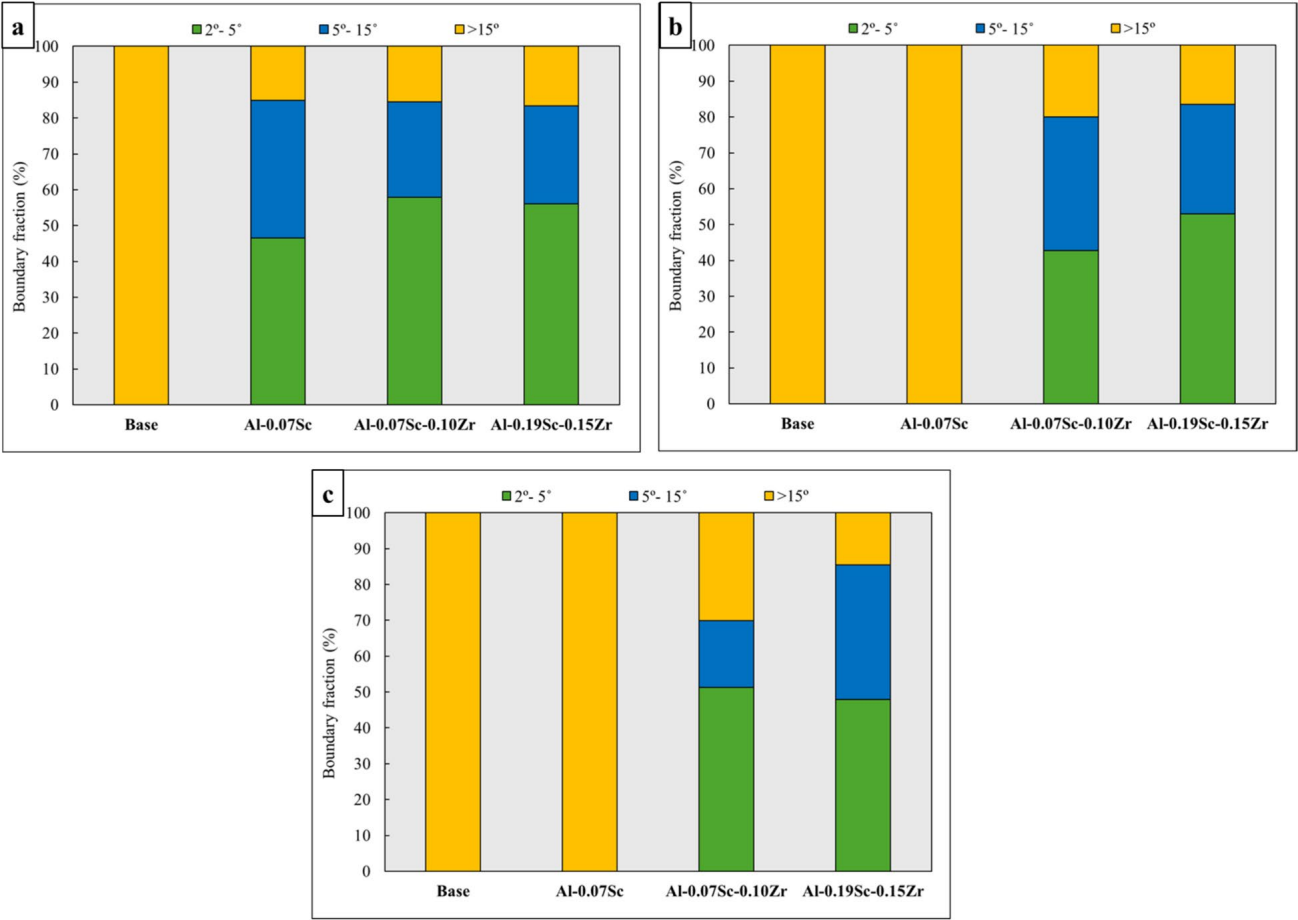
Evolution of misorientation angle distribution of boundaries after post-deformation annealing for 1 h at **a** 500 °C, **b** 550 °C, and **c** 575 °C

Figure 9 shows the All-Euler EBSD maps of the four investigated alloys, revealing the grain structures after post-deformation annealing at three different temperatures (500, 550, and 575 °C). Figure 10 presents the fractions of grain/sub grain boundaries based on the misorientation angle values after post-annealing. The base alloy, after the annealing at 500 °C, exhibited a coarse grain structure, and all grains showed no substructure inside (Fig. 9a), indicating that the static recrystallization had been completed during annealing (Qian et al. 2020). The grain structure was dominated by 100% of high-angle grain boundaries (HAGBs, > 15°) (Fig. 10a). When the annealing temperature increased to 550 °C (Fig. 9b), some small island grains (indicated by the white arrows) were observed to be trapped between growing larger grains, but a similar coarse grain structure consisting entirely of HAGBs remained (Fig. 10b). As the annealing temperature further increased to 575 °C (Fig. 9c), the abnormal grain growth (AGG) became pronounced (Li et al. 2019b) as all grain coarsened dramatically, often reaching several hundred micrometers. The lack of precipitate pinning in the base alloy facilitated the grain growth during high-temperature annealing.

Unlike the base alloy, the Al-0.07Sc alloy exhibited a partially recovered microstructure after the post-annealing at 500 °C (Fig. 9d), characterized by a high fraction of LAGBs (46.5%) and MAGBs (38.3%) (Fig. 10a). Only a few newly formed equiaxed grains appeared along the original grain boundaries (indicated by white arrows), suggesting the commencement of static recrystallization (Qian et al. 2023). As the annealing temperature increased to 550 and 575 °C, the microstructures transformed to fully recrystallized structures (Fig. 9e, f). This was also indicated by the transformation to 100% HAGBs (Fig. 10b, c).

For the Al-0.07Sc-0.10Zr alloy, after annealing at 500 °C, the as-deformed grain structure was retained (Fig. 9g) with 57.8% of the boundaries classified as LAGBs (Fig. 10a). When the annealing temperature increased to 550 °C, the alloy still showed a recovered structure (Fig. 9h) but with some small equiaxed grains at the original grain boundaries (see white arrows), suggesting the onset of static recrystallization at this temperature. As the annealing temperature further increased to 575 °C, partial recrystallization was observed, and large recrystallized grains appeared (white arrow in Fig. 9i). However, despite the occurrence of static recrystallization, the recrystallized fraction at this temperature accounted for only 24.5% of the total area of the microstructure. In comparison, both the base and Al-0.07Sc alloys were completely recrystallized at the same annealing temperature, indicating the Al-0.07Sc-0.10Zr alloy exhibited enhanced resistance to recrystallization and grain growth.

Regardless of the annealing temperature, the Al-0.19Sc-0.15Zr alloy retained a highly stable deformed grain structure characterized by elongated grains subdivided by numerous sub grain boundaries (Fig. 9j–l). The EBSD maps revealed no evidence of new equiaxed recrystallized grains, even at the highest annealing temperature of 575 °C. Quantitatively, the fraction of LAGBs only slightly decreased with increasing temperature from 56% at 500 °C to 53% at 550 °C and further to 48% at 575 °C (Fig. 10a–c). These results confirm that the Al-0.19Sc-0.15Zr alloy exhibited the highest resistance to recovery and recrystallization among the four alloys investigated.

**Fig. 11:**
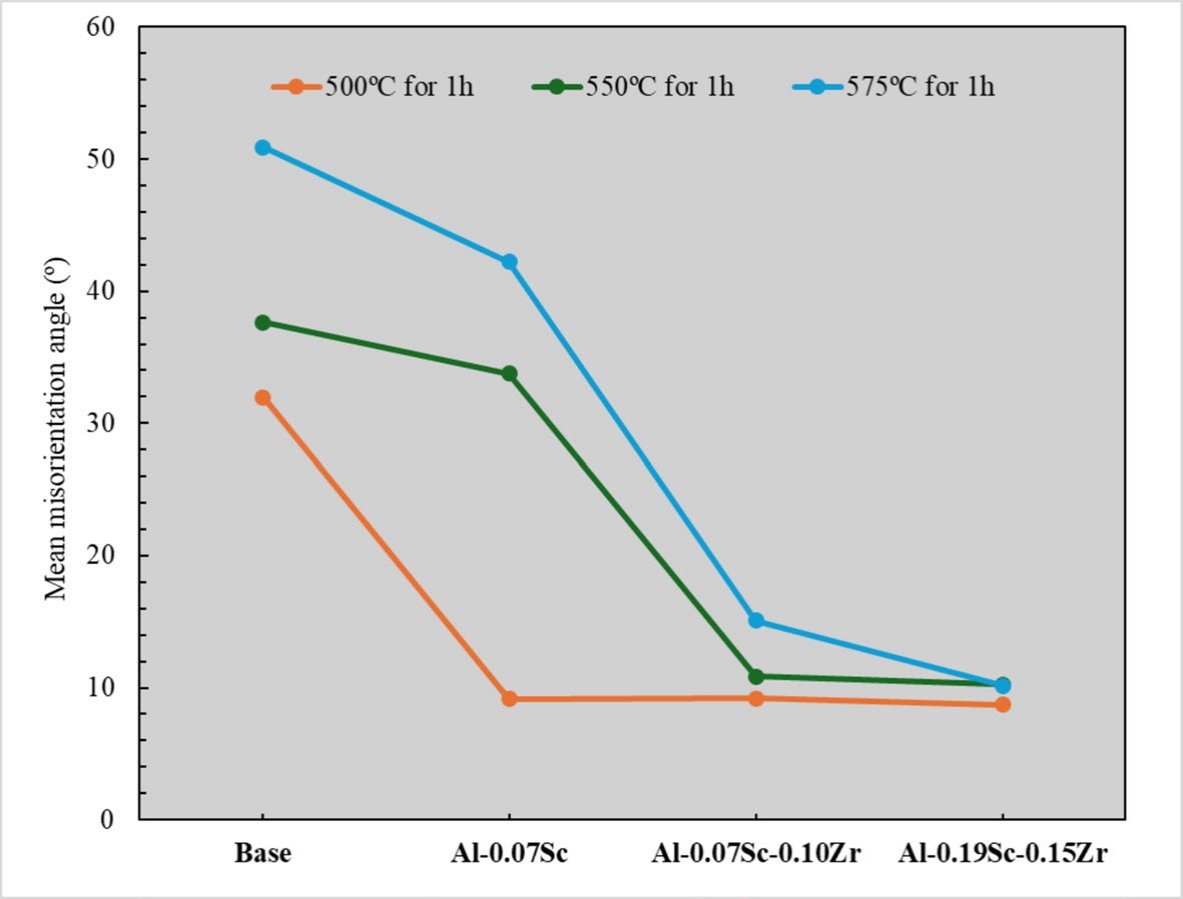
Mean misorientation angles after post-deformation annealing at three different temperatures for the four investigated alloys

To further characterize the evolution of the grain structure during the post-annealing, the mean misorientation angles of the grain boundaries were quantitatively analyzed based on the EBSD data and are presented in Fig. 11. The additions of Sc and Zr decreased the mean misorientation angles at all three annealing temperatures. On the other hand, as the annealing temperature increased, the mean misorientation angles increased in all four alloys. This indicates a higher degree of softening by recovery and recrystallization with rising annealing temperature, aligning with the microstructural observations presented in Fig. 9. After annealing at 500 °C, the base alloy exhibited a mean misorientation angle of 32º. This value increased to 37.6° and 50.9° for the fully recrystallized structures following the annealing at 550 and 575 °C, respectively. On the contrary, after annealing at 500 °C, all three Sc-containing alloys exhibited mean misorientation angles below 10° due to the remaining deformed grain structure. For the Al-0.07Sc alloy, increasing the annealing temperatures to 550 and 575 °C resulted in increasing mean misorientation angles to 33.7° and 42.1°, respectively, showing the progression of the recrystallization process. The combined addition of Sc and Zr demonstrated a clear reduction of the mean misorientation angles, showing an increased recrystallization resistance. For instance, after annealing at the highest temperature of 575 °C, the Al-0.07Sc-0.10Zr alloy exhibited a mean misorientation angle of 15.1° with a partially recrystallized structure while the Al-0.19Sc-0.15Zr alloy showed a lower mean misorientation angle of 10.1°, which further confirms its highest recrystallization resistance among the four alloys studied.

Overall, the EBSD analysis including all Euler EBSD maps, misorientation distributions, and mean misorientation angles provided a comprehensive assessment of the microstructural evolution during post-annealing treatments. The results indicate that microalloying with 0.07 wt.% Sc alone was sufficient to maintain grain stability at 500 °C, but recrystallization occurred at higher temperatures. The co-addition of Zr further enhanced the thermal stability, with the Al-0.19Sc-0.15Zr alloy exhibiting the greatest resistance to recrystallization and grain growth. In contrast, the base alloy exhibited severe microstructural degradation, becoming fully recrystallized and prone to AGG.

## Discussion

### Effect of Al_3_Sc and Al_3_(Sc,Zr) precipitates on recrystallization resistance

The low-temperature homogenization at 350 ºC applied in this study was to promote the formation of Al_3_Sc/Al_3_(Sc,Zr) precipitates to act as recrystallization inhibitors during both hot deformation and post-annealing. TEM observation (Sect. 3.2) confirmed that numerous nanoprecipitates were formed during the low-temperature homogenization in all three Sc-containing alloys. The fine and coherent Al_3_Sc or Al_3_(Sc,Zr) precipitates are known to effectively hinder grain boundary migration and to exert a Zener pinning force, preventing grain growth and recrystallization. The Zener drag pressure (*P*_*z*_), which is the force per unit area exerted by the particles on the grain boundary, can be expressed by Eq. 2 (Humphreys and Hatherly 2012; Eivani et al. 2011; Forbord et al. 2004).

2 $${\mathrm P}_{\mathrm Z}=\frac{3\ast Y_{GB}\ast f_v}{2r}$$

where γ_GB_ is the specific grain boundary energy (0.32 J/m^2^), *r* is the average radius of the particle, and *f*_v_ is the volume fraction of the precipitates, which can be expressed by Eq. 3 (Humphreys and Hatherly 2012).

3 $$f_v=\frac43\mathrm{\pi r}^3\ast{\mathrm N}_{\mathrm d}$$

**Fig. 12:**
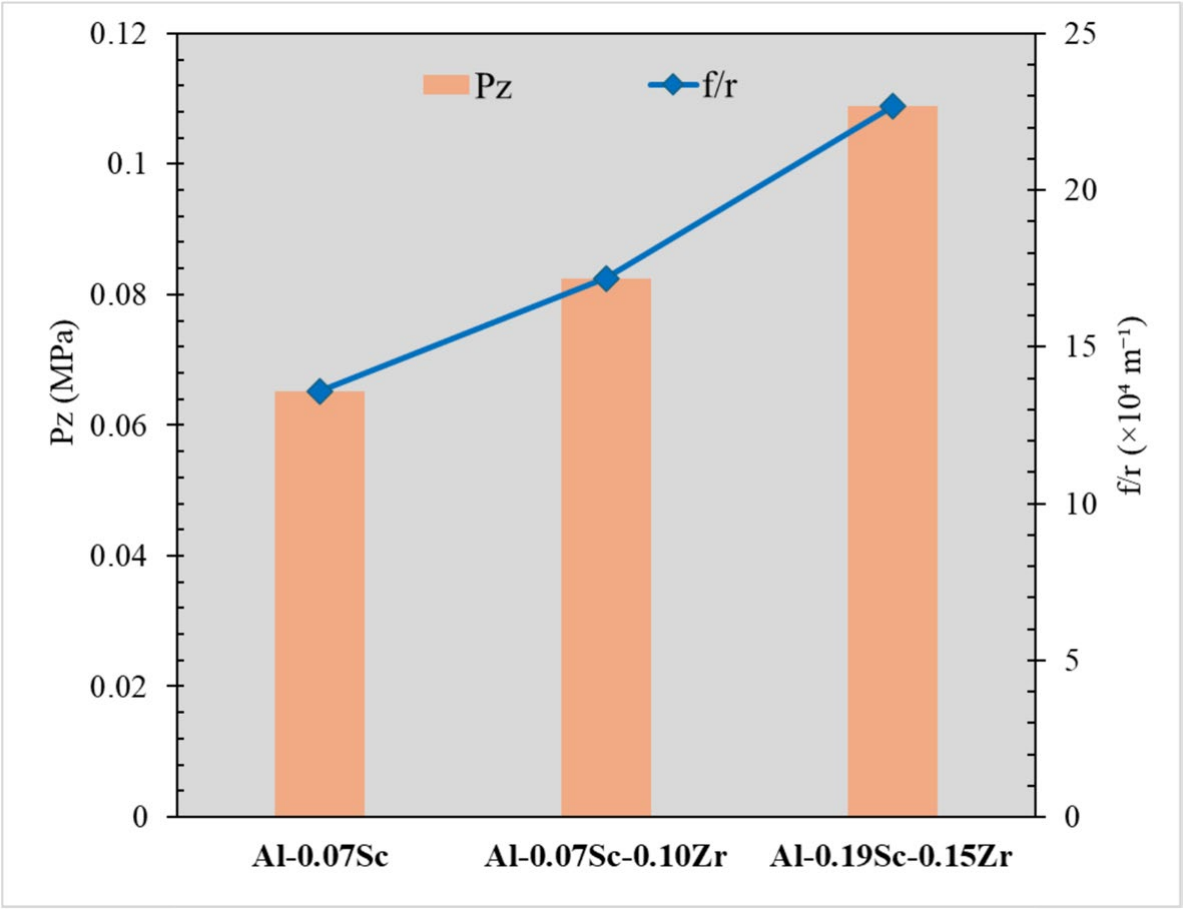
*P*_*z*_ and f_v_/r values of the four investigated alloys after homogenization

The base alloy possessed relatively few particles (just Fe-rich intermetallic particles) without any precipitates, and therefore, its Zener drag pressure, *P*_*z*_, is almost negligible. For the three Sc-containing alloys, as shown in Eq. 2, a high value of f_v_/r is required to achieve a large Zener drag pressure on grain boundary migration to retard the recovery and recrystallization processes (Forbord et al. 2004). *P*_*z*_ can be greatly increased by maximizing the volume fraction and minimizing the size of particles. As shown in Table 2, the Al_3_Sc precipitates formed in the Al-0.07Sc alloy exhibited the coarsest precipitate size and the lowest density among the three Sc-containing alloys, and therefore, it has the lowest volume fraction (Eq. 3). The co-addition of Zr contributed to reducing the size of the precipitates and to increasing their number density and volume fraction. As shown graphically in Fig. 12, the *f*_v_/*r* ratio increased from 13.6 × 10^4^ m^−1^ in the Al-0.07Sc alloy to 17.2 × 10^4^ m^−1^ in the Al-0.07Sc-0.10Zr alloy, and further to 22.7 × 10^4^ m^−1^ in the Al-0.19Sc-0.15Zr alloy. Correspondingly, among the three Sc-containing alloys, the Al-0.07Sc alloy exhibited the lowest *P*_*z*_ of 0.06 MPa, while the *P*_*z*_ value in the Al-0.07Sc-0.10Zr was 0.08 MPa due to the formation of more stable and finer Al_3_(Sc,Zr) precipitates. The Al-0.19Sc-0.15Zr alloy, where the increase of Sc and Zr contents resulted in the highest f_v_/r value, exhibited the highest *P*_*z*_ of 0.11 MPa. The higher *P*_*z*_ values account for the improved grain stability observed during the post-annealing in the two Sc/Zr-containing alloys, as compared to the base alloy and the Al-0.07Sc alloy.

### Evolution of Al_3_Sc and Al_3_(Sc,Zr) precipitates during post-deformation annealing

**Fig. 13:**
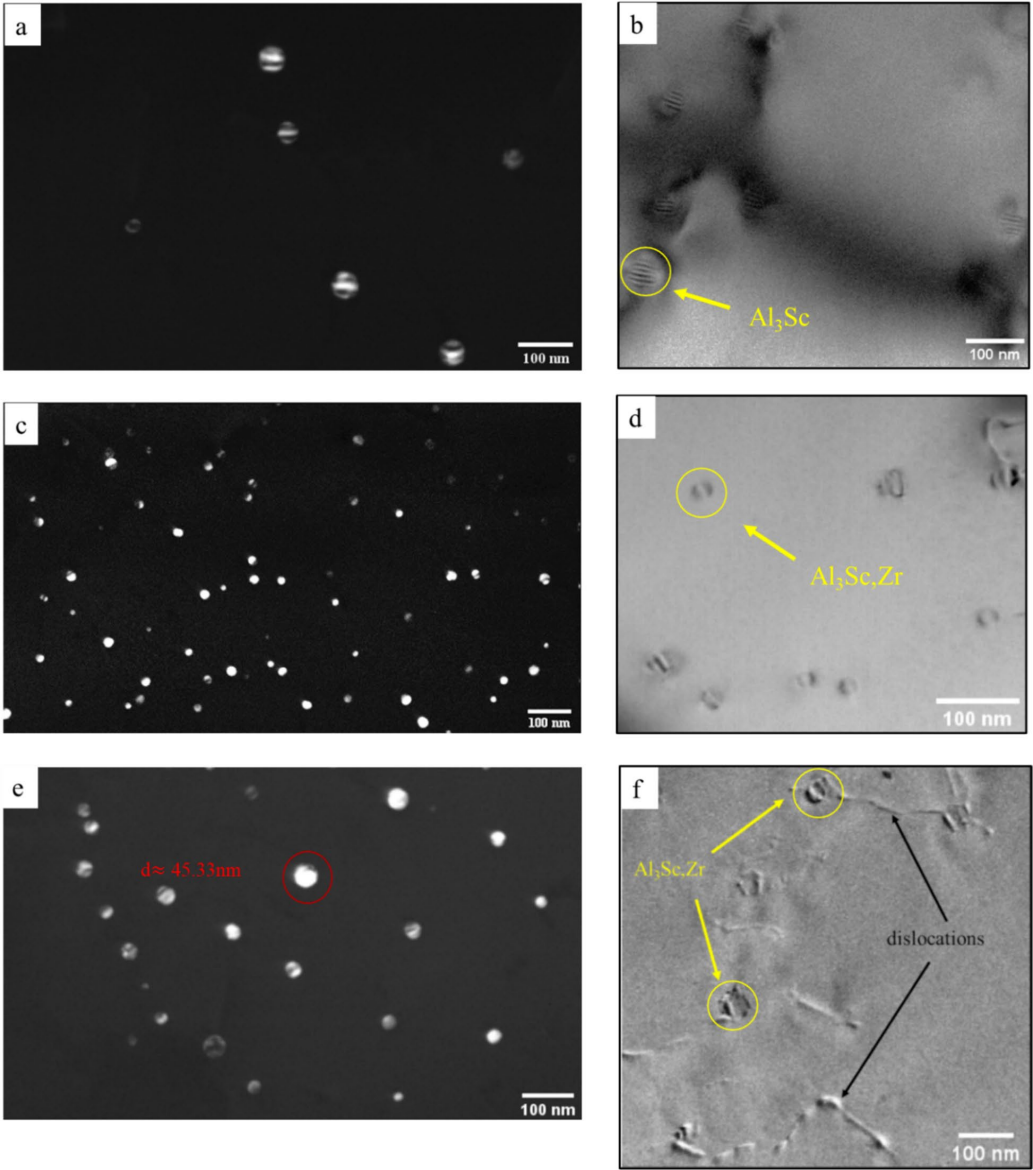
TEM micrographs of the Al_3_Sc/Al_3_(Sc,Zr) precipitates **a**, **b** Al-0.07Sc alloy after annealing at 500 °C for 1 h, **c**, **d** Al-0.07Sc-0.10Zr alloy after annealing at 550 °C for 1 h, and **e**,** f** Al-0.19Sc-0.15Zr alloy after annealing at 575 °C for 1 h

The Zener drag pressure analysis in the previous section highlighted the role of dispersoid size and number density in maintaining grain stability. To further explore the thermal stability of dispersoids, targeted TEM investigations were conducted on selected samples from the three Sc-containing alloys after post-annealing. After annealing at 500 °C, the Al_3_Sc precipitates in the Al-0.07Sc alloy appeared as shown in Fig. 13a, b. The precipitates retained their spherical morphology but grew to an average diameter of 42 ± 4.7 nm, which indicates significant coarsening during post-annealing compared to their average diameter of 6.6 ± 1.9 nm after low-temperature homogenization at 350 °C (Figs. 3a and 4, and Table 2). The presence of Moiré fringes within the precipitates (Fig. 13a) suggests a slight misalignment with the aluminum matrix, resulting from the change in particle size and a gradual loss of coherency (Buranova et al. 2017; Jiang et al. 2020). This observation aligns with the findings of Iwamura et al. (2004), who reported that the critical radius for Al_3_Sc to lose coherency with the matrix ranges between 15 and 40 nm. The start of loss in coherency of those particles is also detected by the presence of interfacial dislocations (appearing as parallel lines observed on the precipitates) in the bright field TEM (Fig. 13b) (Marquis and Seidman 2001). The EBSD result of the Al-0.07Sc alloy after annealing at 500 °C showed a mostly recovered microstructure (Fig. 9d), indicating that these coarsened Al_3_Sc can still successfully resist recrystallization. However, the observed coarsening and partial loss of coherency after the 500 °C treatment indicate a reduced pinning effect of the Al_3_Sc precipitates at higher annealing temperatures. Those TEM observations help to explain the EBSD results, which show that the microstructure was fully recrystallized after annealing at 550 °C and 575 °C (Fig. 9e, f, respectively), confirming the reduced effectiveness of Al_3_Sc precipitates at higher temperatures.

Unlike the Al-0.07Sc alloy, the Al-0.07Sc-0.10Zr alloy showed excellent resistance to recrystallization with a recovered grain structure after the annealing at 550 °C. The TEM images in Fig. 13c, d show the Al_3_(Sc,Zr) precipitates remained relatively stable with a moderate coarsening to an average diameter of 15.5 ± 3.5 nm (Fig. 13c), compared to 5.5 ± 1.3 nm in the homogenized condition (Table 2). The bright-field TEM image in Fig. 13d reveals Al_3_(Sc,Zr) precipitates that exhibited a “bean-like” contrast, a characteristic feature indicating that the coherency between the precipitate and the matrix was maintained (Xu et al. 2019; Iwamura and Miura 2004; Zou et al. 2007). Even after annealing at 550 °C for 1 h, those coherent precipitates can still effectively pin grain boundaries and dislocations, thereby hindering grain growth and recrystallization (Xiang et al. 2016).

The enhanced recrystallization resistance observed with Zr co-additions in our results aligns with previous studies that have reported a delayed onset of recrystallization in Al-Sc-Zr alloys compared to Al-Sc alloys. For instance, Zakharov et al. (2014) found that a hot-pressed Al-0.2% Sc strip began recrystallizing at 490 °C and was fully recrystallized at 550 °C, whereas in the alloy containing Zr, recrystallization started at 570 °C and ended at 600 °C. Similar observations were also reported by Jia et al. (2012), who found that the recrystallization onset temperature for a binary extruded Al-Sc alloy was 520 °C, while the addition of Zr increased the onset temperature by 100 °C.

However, increasing the annealing temperature further to 575 °C resulted in the appearance of large recrystallized grains in the recovered microstructure of the Al-0.07Sc-0.10Zr alloy (Fig. 9i), suggesting a localized loss of pinning pressure owing to coarsening of the precipitates at this high temperature. As the precipitates grew, their effectiveness in pinning grain boundaries diminished, allowing some regions to undergo recrystallization (Ocenasek and Slamova 2001; Rudnizki et al. 2010).

The Al-0.19Sc-0.15Zr alloy was the only alloy retaining the mostly deformed microstructure after all the three post-annealing treatments, even at the highest temperature of 575 °C. The TEM micrographs of the Al_3_(Sc,Zr) precipitates after annealing at 575 °C are displayed in Fig. 13e, f. The precipitates exhibit a wide size distribution, with an average diameter of approximately 24.9 ± 8.9 nm and a few large ones reaching the maximum diameter of 45.3 nm (Fig. 13e). At such a high temperature, the precipitates underwent obvious coarsening compared to their initial size after homogenization. However, most of the precipitates were still found to be coherent. The bright-field TEM image of Fig. 13f illustrates the bowing of dislocations at their interaction points with the Al_3_(Sc,Zr) precipitates, which exerted a pinning effect and acted as obstacles that impede dislocation motion (Babaniaris et al. 2020; Leng et al. 2021). It is apparent that Al_3_(Sc,Zr) precipitates in the Al-0.19Sc-0.15Zr alloy exhibited a sufficient thermal stability even during annealing at 575 ºC to effectively inhibit recrystallization and grain growth and maintaining a superior grain stability.

The thermal stability of Al_3_(Sc,Zr) precipitates in some aluminum alloys with similar Sc and Zr contents (approximately 0.2 wt.% Sc and 0.15 wt.% Zr) has been documented in the literature. For instance, Deng et al. (2013) observed that in a rolled Al-Zn-Mg alloy containing 0.25 wt.% Sc and 0.1 wt.% Zr, the Al_3_(Sc,Zr) particles remained coherent after annealing at 600 °C for 1 h. Lee et al. (2002) reported that an Al–Mg–0.2Sc–0.12Zr alloy, processed by equal-channel angular pressing, exhibited excellent grain stability when subjected to annealing at up to 550 °C. The improved grain stability observed in Sc/Zr-containing alloys at high temperatures highlights their strong potential for high-temperature applications like brazed heat exchangers, where maintaining grain structure and preventing excessive grain growth are crucial.

The influence of thermally stable dispersoids, formed during homogenization, is evident across all subsequent processing stages from hot deformation to post-annealing. Table 3 summarizes and compares the overall behavior of the four investigated alloys by presenting the calculated Zener drag pressure *P*_*z*_ (derived from dispersoid characteristics in the as-homogenized condition), the flow stress values (from hot compression tests), and the AGG behavior observed after annealing at 500, 550, and 575 °C. This highlights how the initial dispersoid stability influences the deformation behavior and microstructural evolution across all key processing steps. Alloys containing both Sc and Zr exhibited higher flow stresses and reduced flow softening compared to the Sc-only and base alloys (Fig. 5), indicating suppressed dynamic recovery (Babaniaris et al. 2020). This observation is also supported by the microstructural evolution observed in EBSD analysis of the as-deformed samples (Figs. 6 and 7). The fractions of LAGBs and MAGBs in the two Sc/Zr-containing alloys were greatly increased to retain the deformed structures relative to the Sc-only alloy (Al-0.07Sc) owing to the presence of finer and more numerous Al_3_(Sc,Zr) precipitates formed during homogenization. Those precipitates remained stable during the hot deformation (Fig. 8).

**Table 3:** Summary of Zener drag pressure (*Pz*​), flow stress at 450 °C, and observed AGG behavior after annealing at 500 °C, 550 °C, and 575 °C for 1 h

Alloy	*Pz *(MPa)	Flow Stress (MPa)	AGG at 500 °C/1h	AGG at 550 °C/1h	AGG at 575 °C/1h
Base	0	27	Severe	Severe	Severe
Al-0.07Sc	0.06	36.5	None	Moderate	Severe
Al-0.07Sc-0.10Zr	0.08	42.3	None	None	Moderate
Al-0.19Sc-0.15Zr	0.11	53.5	None	None	None

Following the post-annealing heat treatments, the two Sc/Zr-containing alloys exhibited significantly enhanced grain stability. The migration of the grain boundaries was strongly hindered at all three annealing temperatures. This behaviour is attributed to the higher *P*_*z*_ values in the Sc/Zr containing alloys compared with the Al-0.07Sc alloy. Consequently, the two Sc/Zr-containing alloys display a remarkably higher recrystallization resistance.

From an economic perspective, the amount of Sc addition is a key factor, as increasing the Sc content significantly raises the product costs. In this regard, the Al-0.07Sc-0.10Zr alloy remains a viable option in applications where the effective thermal exposure does not exceed about 1 h at 550 °C, thereby balancing cost-effectiveness with performance. Yet in more severe scenarios where the effective thermal exposure is above 1 h at 575 °C, the Al-0.19Sc-0.15Zr alloy might be a good choice to ensure better grain stability. However, as shown in Fig. 5, the flow stress also increases with increasing Sc/Zr content. This will result in a decreased extrudability, which can also increase the overall cost. Possible options to decrease the flow stress and improve the extrudability might be the use of a higher hot deformation temperature or a modified homogenization treatment.

## Conclusions

This study investigated the effects of Sc and Zr microalloying on the recrystallization behavior and grain growth of four 1xxx aluminium heat exchanger alloys during three post-deformation annealing treatments at 500, 550, and 575 °C for 1 h to simulate the high-temperature brazing process. The following conclusions are drawn from the main results:

1. A low-temperature homogenization at 350 °C for 24 h promoted the formation of numerous Al_3_Sc/Al_3_(Sc,Zr) nanoprecipitates in the three Sc-containing alloys. With increasing contents of Sc and Zr, the average size of these precipitates decreased, and the number density increased. During hot compression deformation, all four alloys underwent dynamic recovery. Compared to the base alloy, the three Sc-containing alloys exhibited higher fractions of low-angle grain boundaries, indicating that the microalloying with Sc and Zr imposed large restrictions on the recovery process.
2. Following the post-deformation annealing at 500 °C, full recrystallization occurred in the base alloy. Increasing the annealing temperature resulted in the occurrence of abnormal grain growth due to the lack of precipitates. In the Al-0.07Sc alloy, the Al_3_Sc precipitates effectively retarded recrystallization during annealing at 500 °C, while full recrystallization occurred at the higher annealing temperatures.
3. The Al-0.07Sc-0.10Zr alloy preserved its mostly deformed and recovered microstructure during annealing up to 550 °C owing to the stable Al_3_(Sc,Zr) precipitates, which exerted a high Zener drag pressure on grain boundary migration that retards the recovery and recrystallization. Local recrystallization occurred after annealing at the higher temperature of 575 °C.
4. The Al-0.19Sc-0.15Zr alloy preserved its mostly deformed microstructure even after annealing at 575 °C, showing the highest recrystallization resistance among the four alloys studied, owing to its highest number density and finest size of Al_3_(Sc,Zr) precipitates.
5. The Al-0.07Sc-0.10Zr alloy provides a good balance between cost-effectiveness and grain stability, making it suitable when the effective thermal exposure during brazing or other elevated temperature processing does not exceed the equivalent of about 1 h at 550 °C. However, under extreme conditions where the effective thermal exposure is equivalent to 1 h at 575 °C, the Al-0.19Sc-0.15Zr alloy may be more appropriate.

## Data Availability

The data are available from the corresponding author upon reasonable request.
